# Codeine and Metabolite Concentrations in the Breastfed Neonate

**DOI:** 10.1002/pan.70227

**Published:** 2026-05-21

**Authors:** Brian J. Anderson, Jacqueline A. Hannam

**Affiliations:** ^1^ Department of Anaesthesiology University of Auckland Auckland New Zealand; ^2^ Department of Pharmacology University of Auckland Auckland New Zealand

**Keywords:** breastmilk, codeine, compartment model, drugs, morphine, pharmacodynamics, pharmacokinetics, pharmacology

## Abstract

Analgesic effect from codeine is from its metabolite, morphine. Morphine is formed by the O‐demethylation of codeine and that enzyme is controlled by the cytochrome P450 2D6. More than 60 alleles in the CYP2D6 gene have been identified. This spectrum of polymorphism can be categorized into four groups: poor (PM), intermediate (IM), normal (NM), and ultra‐rapid (UR) metabolizers. Codeine is rarely used in children because of fears that those with the UR genotype may suffer respiratory depression from increased morphine production. There is also concern that postpartum women medicated with codeine and who are UR metabolizers may have enough morphine in breastmilk to cause respiratory depression in a breastfed neonate. A pharmacokinetic compartment model was used to explore this assumption. There are five issues to consider in the pharmacological pathway from maternal ingestion of codeine to neonatal morphine plasma concentration: maternal morphine concentration that is dependent on genotype, milk to plasma concentration ratio, neonatal codeine and morphine exposure from breastmilk, codeine metabolism to morphine in the neonate, and morphine clearance in the neonate. The compartment model confirmed the implausibility of neonatal opioid toxicity from breastfeeding. Currently, short‐term maternal use of prescription opioids (other than codeine) is considered safe and infrequently presents a hazard to the newborn. Postpartum women are denied codeine for analgesia and yet predicted neonatal morphine concentrations are lower than 1 μg/L, regardless of genotype. Maternal opioids are used with caution, especially after multiple doses, and neonates younger than 46 weeks postmenstrual age are observed for drowsiness and respiratory depression. The maternal use of codeine requires similar considerations and the current ban on codeine use in postpartum women who are breastfeeding requires review.

## 1 Introduction

The usefulness of codeine as an analgesic for children has long been debated [1]. Codeine itself is a weak analgesic. Analgesic effect is gained from codeine's metabolite, morphine. There are concerns that codeine might be either an ineffective analgesic or too effective and contribute to respiratory depression in those with CYP2D6 polymorphisms for the formation clearance of its metabolite, morphine [2]. Codeine is no longer recommended for pain management in infants and children because of deaths when it was prescribed to children with CYP2D6 ultra‐rapid (UR) polymorphism. Most children's hospitals removed this drug from their formulary because of safety concerns [1, 3, 4, 5, 6].

Codeine was always considered a reasonable analgesic for postpartum women. Morphine concentrations in the breastfed neonate had been reported to range from < 0.5 to 2.2 μg/L after mothers were given 240 mg/day in divided doses before the turn of the century (1997) [7]. However, disquiet around the use of codeine in postpartum women who were breastfeeding increased following a report in 2006 of a neonate who died, assumably due to breastmilk ingestion that contained high concentrations of morphine [8]. Although numerous investigators queried the feasibility of the neonatal morphine plasma concentration of 70 μg/L after maternal codeine ingestion [9, 10, 11] and even the veracity of the report [12], breastfeeding women continue to be denied codeine for analgesia [13]. The senior author of reports concerning neonatal opioid toxicity after breastfeeding [8, 14, 15, 16] has since been exposed as fraudulent [17]. This has created some puzzlement in the pediatric anesthesia community about the pharmacology related to drugs contained in breastmilk and neonatal impact, particularly for codeine. Although short‐term maternal use of prescription opioids (other than codeine) is considered safe and may infrequently present a hazard to the newborn, opioids should still be used with caution, especially after multiple maternal doses with breastfed neonates younger than 46 weeks postmenstrual age. Those neonates should be observed for drowsiness and respiratory depression [13].

## 2 Pharmacology Considerations

Some clarity to the importance of CYP2D6 polymorphisms can be gleaned through simulation using pharmacokinetic compartment modeling [18]. The compartment model needed to address five issues of imprtance [19].

### 2.1 Dose to Mother

The pharmacokinetics of codeine can be described using a one‐compartment model with a volume of distribution (V 3–6 L/kg) and clearance (CL 10–15 mL/min/kg) [20]. Codeine is absorbed from the gastrointestinal tract at a rate described by the absorption rate constant (*K*_a_ = 4–8 h^−1^). The drug has a high bioavailability (*F*_CODEINE,ADULT_ = 0.85) [18]. Codeine displays linear kinetics and codeine concentration relates directly to dose.

**FIGURE 1:**
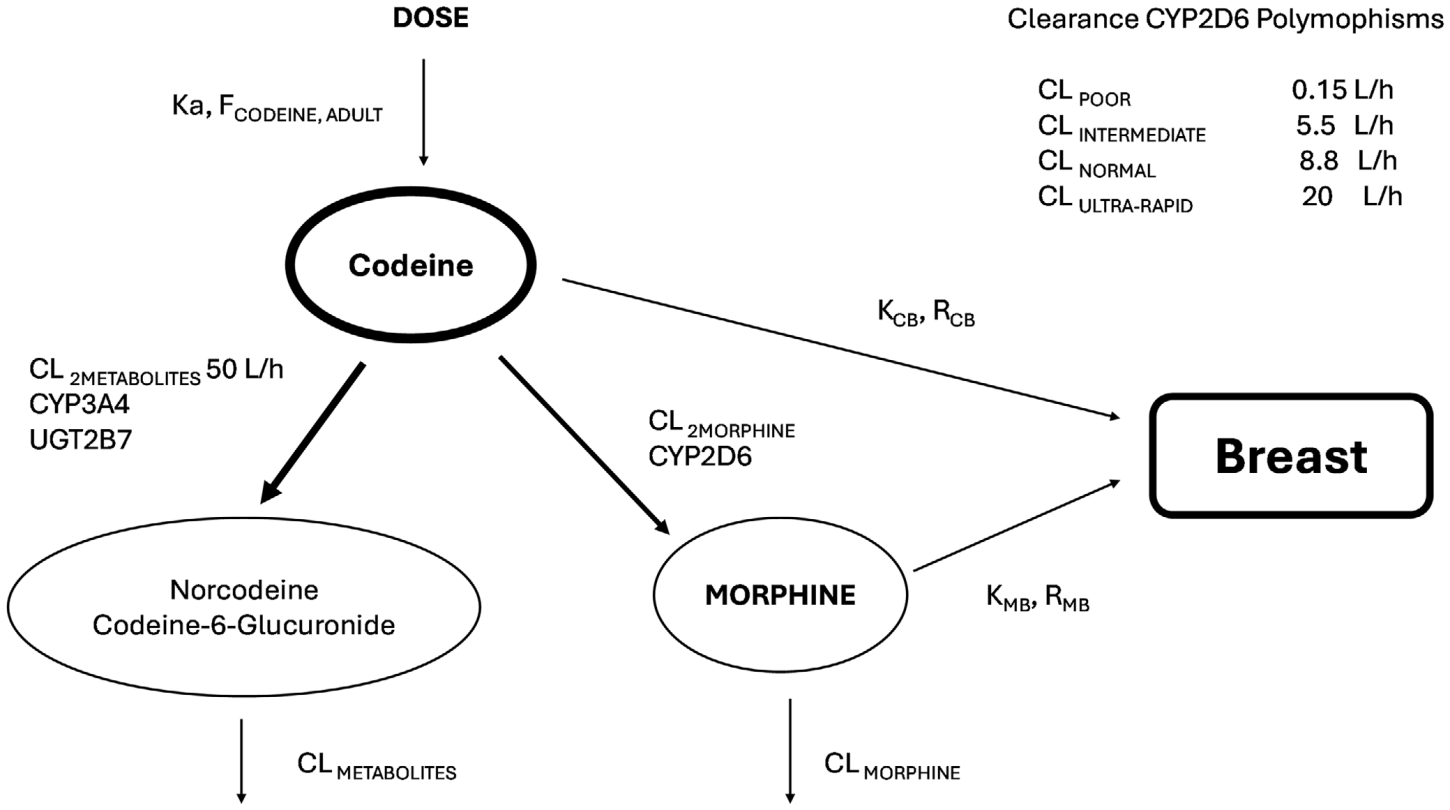
A schematic demonstrating codeine metabolism with codeine and morphine equilibration in breastmilk using a compartment model in an adult. *F* is relative bioavailability; *K*_a_ is the absorption rate constant; *K*_CB_ is the equilibration rate constant for transfer of codeine from plasma to milk; *K*_MB_ is the equilibration rate constant for transfer of morphine from plasma to milk; *R*_CB_ is the milk to plasma concentration ratio for codeine while *R*_MB_ is that for morphine; CL is clearance.

Most codeine undergoes glucuronidation (80%) to an inactive metabolite, codeine‐6‐glucuronide by the enzyme UGT2B7. Another inactive metabolite, norcodeine, accounts for a smaller amount (10%–15%) by N‐demethylation (CYP3A4). Codeine is a prodrug and a further 5%–15% is converted into morphine by hepatic O‐demethylation (CYP2D6) [20]. Metabolites such as codeine‐6‐glucuronide, norcodeine, and the morphine metabolites (morphine‐3‐glucuronide and morphine‐6‐glucuronide) are water soluble and are cleared by the kidney. The total clearance of codeine is dependent on clearance to codeine‐6‐glucuronide and norcodeine with a further variable clearance to morphine that is dependent on CYP2D6 polymorphisms. A compartment model for codeine metabolism is shown in Figure 1. Morphine has a 200‐fold higher affinity for opioid receptors than codeine and is the dominant source of analgesia after taking codeine orally.

**FIGURE 2:**
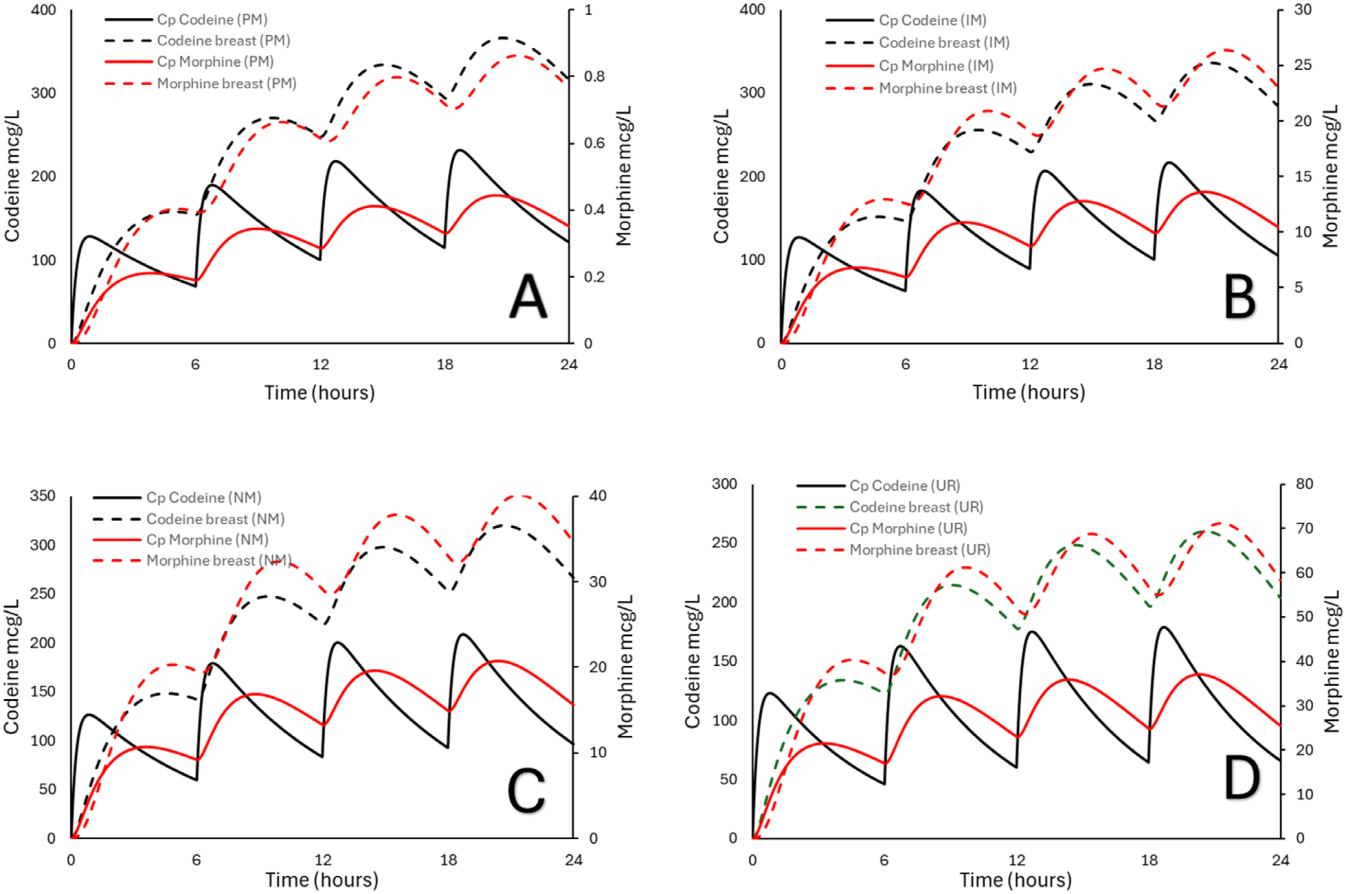
Simulated codeine and morphine plasma concentrations and milk concentrations in a breastfeeding woman given codeine 60 mg four times a day. (A) Concentrations in a breastfeeding woman (Day 5) who is a poor metabolizer (PM). Concentrations in (B) an intermediate metabolizer (IM), (C) a fast metabolizer (NM), and (D) an ultra‐fast metabolizer (UF). Cp is plasma concentration, Codeine breast is breastmilk concentration of codeine, Morphine breast is breastmilk concentration of morphine. Parameter estimates for the compartment model are described in the text.

More than 60 alleles in the CYP2D6 gene have been identified [21]. This spectrum of polymorphism can be categorized into four groups; poor (PM), intermediate (IM), normal (NM) and UR metabolizers with corresponding activity ratios of < 0.03, 0.03–1.25, 1.25–2.25, and > 2.25, respectively [18, 22, 23]. Simulations used a high formation clearance (O‐demethylation) rate of 20 L/h for those with the UR genotype in order to explore possible morphine concentrations in the breastfeeding mother and neonate. Consequently, we might anticipate a morphine plasma concentration after oral codeine 60 mg four times daily that are delayed compared to codeine concentrations and approximate to 0.4 μg/L (PM), 10 μg/L (IM), 18 μg/L (NM), and 30 μg/L (UR) at steady state (Figure 2).

### 2.2 Milk to Plasma Concentration Ratio

Plasma codeine concentrations equilibrate (described by rate constant, *K*_CB_) with breastmilk and this occurs approximately 1–2 h after an oral codeine dose. Drug concentrations in breastmilk are typically measured using whole milk samples, as this most accurately reflects the dose the infant ingests. That concentration is higher than plasma concentrations and relates to codeine's p*K*a (8.2), lipophilicity, and protein binding. Codeine is a weak base and is susceptible to ion trapping in breastmilk, which is slightly more acidic (pH ~7.1–7.2) than maternal plasma (pH ~7.4). Codeine ionization makes it harder for it to diffuse back into the plasma, resulting in milk concentrations that are higher than those in the plasma. Milk to plasma ratios differ for each drug and are dependent on physiological and chemical factors [24]. The milk to plasma ratio (*R*_CB_) for codeine is 1.35–2.5, while that for morphine (*R*_MB_) is 0.84–2.5.

Based on a milk to plasma ratio (*R*_CB_) for codeine and morphine of 2, the concentration of codeine in breastmilk after oral maternal ingestion of codeine 60 mg four times daily is approximately 280 μg/L at steady state (Figure 2). The codeine concentration in those UR metabolizers is less (230 μg/L) because total plasma codeine clearance is greatest in that group. Morphine concentration in breastmilk is related to CYP2D6 activity: 0.8 μg/L (PM), 23 μg/L (IM), 35 μg/L (NM), 65 μg/L (UF).

### 2.3 Neonatal Exposure

Although breastmilk concentrations of codeine and morphine are higher than those in maternal plasma, the dose administered to the neonate is dependent on the volume of milk ingested during feeding. A term neonate drinks about 350–550 mL/day in the first week of postnatal life. The stomach is small and gastric emptying delayed. If we assume three hourly feeding of 65 mL in a 5‐day‐old term neonate, then the codeine dose is 18 μg for each feed or 145 μg/day (15 or 120 μg/day in UF metabolizers). Morphine dose is small; 0.5 μg per breastfeed (4 μg/day) (PM), 1.5 μg (12 μg/day) (IM), 2.3 μg (18 μg/day) (NM), 4.3 μg (34 μg/day) (UF). Neonatal exposure is further reduced by first‐pass metabolism, but bioavailability is greater in neonates than in adults because clearance pathways are immature, allowing more active drug to bypass the liver. Although clearance to morphine from codeine is reduced (10% of adult rate), the bioavailability of morphine (*F*_MORPHINE,NEO_ 0.4–0.6) in a neonate is higher than that in an adult compared to that for an adult (*F*_MORPHINE_,_ADULT_ 0.20–0.40) [25].

### 2.4 Neonatal Metabolism of Codeine

Microsomal enzyme activity responsible for hepatic clearance can be classified into three groups [26]: mature at birth but decreasing with age (e.g., CYP3A7 responsible for methadone clearance in neonates), mature at birth and sustained through to adulthood (e.g., plasma esterases that clear remifentanil), or immature at birth. The majority of microsomal enzyme activity is reduced or absent in the neonate. Codeine and metabolite clearance (CYP3A4, CYP2D6, UGT2B7) [27, 28] and renal elimination [29] pathways are immature in the neonate. Neonatal clearance of codeine is small and while only 5%–15% of that total clearance is attributed to CYP2D6 in adults, cytochrome activity is immature in neonates. Review of tramadol metabolism demonstrates its difficulty to discriminate developmental changes in CYP2D6 activity in the first week of life [30, 31]. Breastmilk containing codeine is certainly metabolized to morphine through hepatic O‐demethylation (CYP2D6) in the neonate, but formation clearance is slow.

### 2.5 Neonatal Clearance

**FIGURE 3:**
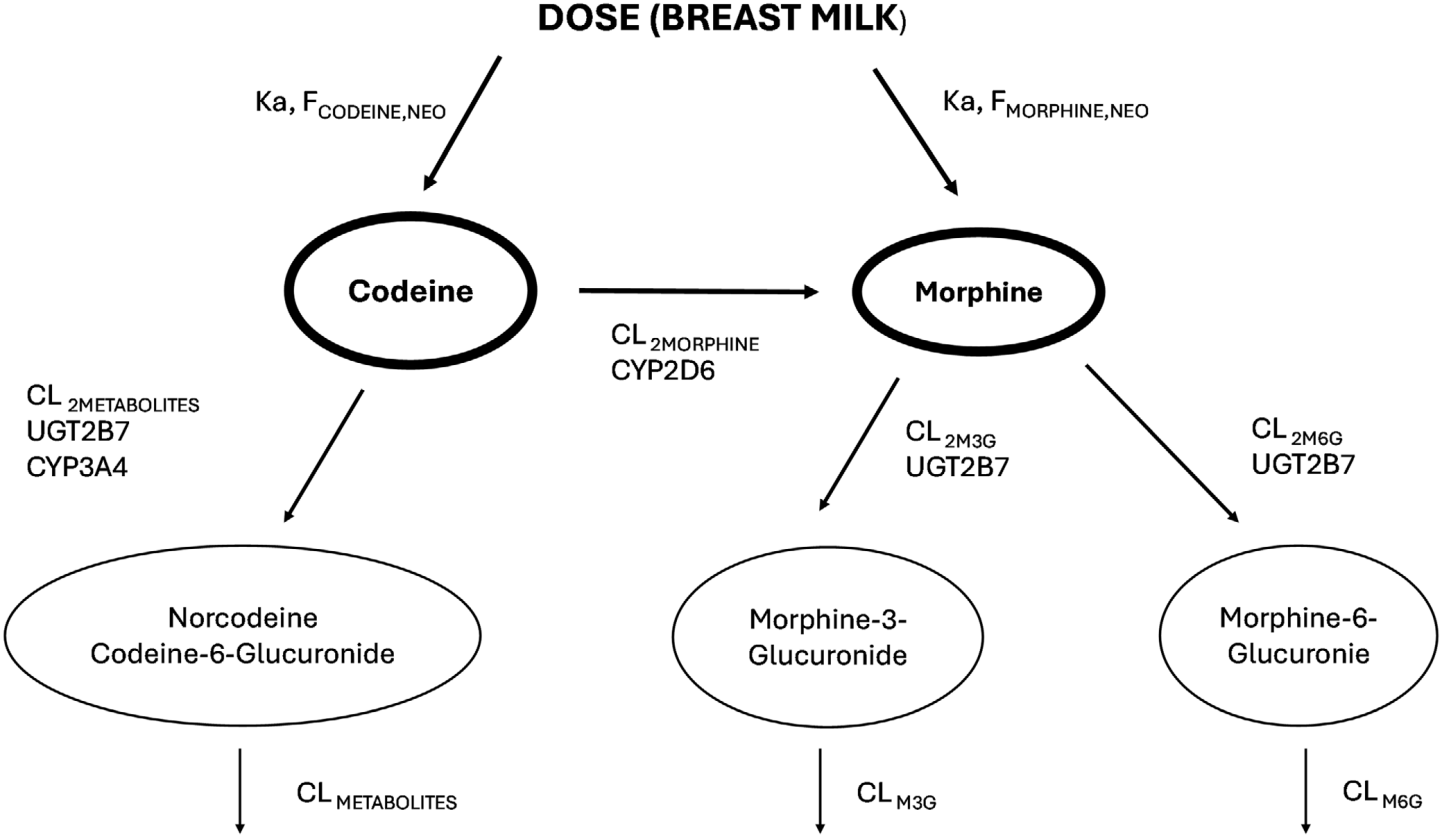
A schematic demonstrating the major metabolic pathways for ingested milk containing codeine and morphine in the neonate. *F* is relative bioavailability; *K*_a_ is the absorption rate constant; CL is clearance.

Neonatal plasma concentrations of any drug are dependent on dose administered and clearance of that drug in the neonate. The neonate ingests both codeine and morphine from a breastmilk feed. There are two sources for the observed morphine concentrations in the neonate (Figure 3) because codeine is metabolized to morphine through hepatic O‐demethylation (CYP2D6), albeit slowly. Morphine concentrations observed in the neonate after maternal codeine are approximately twice those anticipated than those from morphine alone in breastmilk at steady state. This is because codeine in breastmilk is metabolized by the neonate to morphine. That formation clearance is typically slow and was set at 10% adult in the current scenario.

**FIGURE 4:**
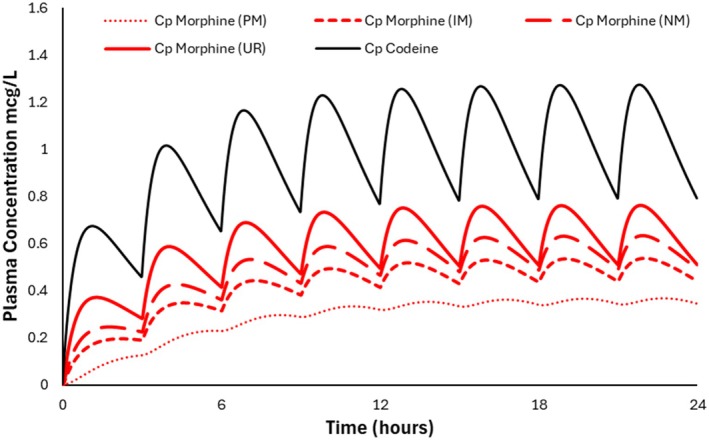
Simulated codeine and morphine plasma concentrations (Cp) in a term breastfeeding neonate of 5 days' age. The mother had been given 60 mg codeine four times a day. Morphine plasma concentrations for a poor metabolizer (PM), an intermediate metabolizer (IM), a fast metabolizer (NM), and an ultra‐fast metabolizer (UF) are shown. Parameter estimates for morphine pharmacokinetics were taken from Bouwmeester NJ. *Br J Anaesth* 2004;92:208–17.

Clearance of water‐soluble metabolites is through the kidney, and renal function is also immature [29]. Consequently, we might anticipate a mean neonatal codeine concentration of approximately 1 μg/L in the neonate at steady state, perhaps lower in the neonate whose mother is an UR metabolizer. Morphine concentrations will be in the range of 0.3–0.7 μg/L irrespective of CYP2D6 genetic polymorphism (Figure 4).

## 3 Discussion

Predicted mean plasma codeine concentration at steady state (1 μg/L) in a 5‐day neonate breastfed by a mother given codeine 240 mg daily was consistent with those reported in term neonates breastfed for 3 days after vaginal delivery (0.8–4.5 μg/L) [7]. Morphine concentrations were also similar to observations in that cohort (0.5–0.8 μg/L) [7]. Neonatal concentrations of morphine lower than 1 μg/L are unlikely to cause respiratory depression. These concentrations are two orders of magnitude lower than those reported in the infant who died after breastfeeding in a mother given codeine for analgesia (70 μg/L) [8]. These low neonatal morphine concentrations were determined in a simulation using compartment models where UR metabolism was assumed faster than usual. These findings confirm an earlier physiology‐based pharmacokinetic (PBPK) model [10] that showed the requirement of combinations of extreme PK parameters to simulate the case report of a neonate who died due to breastmilk ingestion that assumably contained high concentrations of morphine [8]. That analysis implied that an average infant with average PK parameters is unlikely to get toxic [10].

Despite reviews claiming the implausibility of those high morphine concentrations in the neonate being attributable to maternal codeine ingestion [9, 10, 11], a single report of a neonatal morphine concentration (70 μg/L) [8] changed analgesic management in breastfeeding mothers. The report also led to concerns about other analgesic drugs such as tramadol. Tramadol is another drug metabolized to an active metabolite by CYP2D6 polymorphisms [32]. There is a reluctance to use tramadol in breastfeeding mothers for fear of adverse effects in the neonate, despite no evidence that plasma tramadol or its active metabolite (O‐desmethyltramadol) concentrations are high in neonates [33].

Breastfeeding mothers who are given morphine as analgesia are usually given titrated doses to achieve a similar target concentration range to children (10–20 μg/L). Consequently, breastmilk concentrations will approximate 35 μg/L (morphine milk to plasma ratio, *R*_MB_ = 0.8–2.5) in a normal metabolizer and neonatal morphine concentrations of 0.3–0.4 μg/L. These neonates are routinely observed for drowsiness and respiratory depression. If codeine were to be used in breastfeeding mothers, then breastfed neonates would also require observation because morphine concentration in the neonate is twice that observed after morphine alone given to a breastfeeding mother.

The simulation used to predict codeine and morphine concentrations in maternal plasma, breastmilk, and neonatal plasma demonstrated concentrations consistent with those reported by others [7, 34]. Morphine concentrations in children given a weight‐scaled codeine dose might be expected to be similar to those in maternal plasma determined in these current simulations. Plasma morphine concentrations in children given codeine who are UR metabolizers could cause respiratory depression, particularly in those with obstructive sleep apnea [35]. Morphine plasma concentrations greater than 20 μg/L in children are associated with increased respiratory depression. A concentration that produces half morphine's maximum respiratory depressant effect (*C*_50_) of 10–18 μg/L [36, 37] is consistent with clinical observations for both analgesic concentrations (10–20 μg/L) [38, 39] and respiratory depression (hypercapnia in 46% children with concentration > 15 μg/L) [40]. Morphine concentrations of 17–30 μg/L were found in three postoperative deaths associated with repeated‐dose codeine used in the setting of adenotonsillectomy and those children were classified as ultra‐fast metabolizers [41].

The pharmacokinetic parameters (clearance, volume) used to describe both morphine and codeine disposition are associated with considerable between‐subject variability (CV 40%–60%) [42, 43, 44] and plasma concentrations greater than 30 μg/L are possible in an individual child given codeine. However, these simulated plasma concentrations are based on a CYP2D6 activity score where O‐demethylation contributes approximately 30% of the total codeine clearance rather than the recognized 5%–15% [20] of total codeine clearance. Dose remains the major predictor of plasma morphine concentration in children given codeine, and accidental administration of the wrong dose remains the most common drug error in pediatric anesthesia practice [45, 46, 47]. The genotype for CYP2D6 polymorphism may contribute to high metabolite concentrations, but phenotype does not always closely match genotype [31, 48] and other contributors to toxicity should be reviewed. Tramadol, a non‐opioid analgesic, also undergoes O‐demethylation by the polymorphic hepatic enzyme CYP2D6. Tramadol dose, rather than its major active metabolite, O‐desmethyltramadol (M1), remains the major determinant of toxicity because an adult ophthalmic tramadol formulation (100 mg/mL) was used inappropriately for analgesia in children, and dosing errors were not uncommon [49].

A pharmacokinetic model that considered aspects (maternal dose, milk to plasma concentration ratio, neonatal exposure, and neonatal clearance pathways) relating to neonatal drug concentration from breastmilk after maternal drug ingestion supports guidelines for anesthesia and sedation for breastfeeding women [13]. Anesthetic and non‐opioid drugs are transferred to breastmilk in only very small amounts with no evidence of effect on the breastfed infant. Although short‐term maternal use of prescription opioids is usually safe and infrequently presents a hazard to the newborn [19], opioids (and benzodiazepines) should be used with caution, especially after multiple doses and neonates younger than 46 weeks postmenstrual age observed for drowsiness and respiratory depression. The use of codeine in breastfeeding women that is currently banned requires review.

## Funding

This work was funded from institutional resources.

## Conflicts of Interest

The authors declare no conflicts of interest.

## Data Availability

The authors have nothing to report.
